# Supplementary figures and images for: A role of hypoxia-inducible factor 1 alpha in Murine Gammaherpesvirus 68 (MHV68) lytic replication and reactivation from latency
Source: PLoS Pathog. 2019 Dec 6;15(12):e1008192. doi: 10.1371/journal.ppat.1008192 (PMC6975554; doi:10.1371/journal.ppat.1008192)

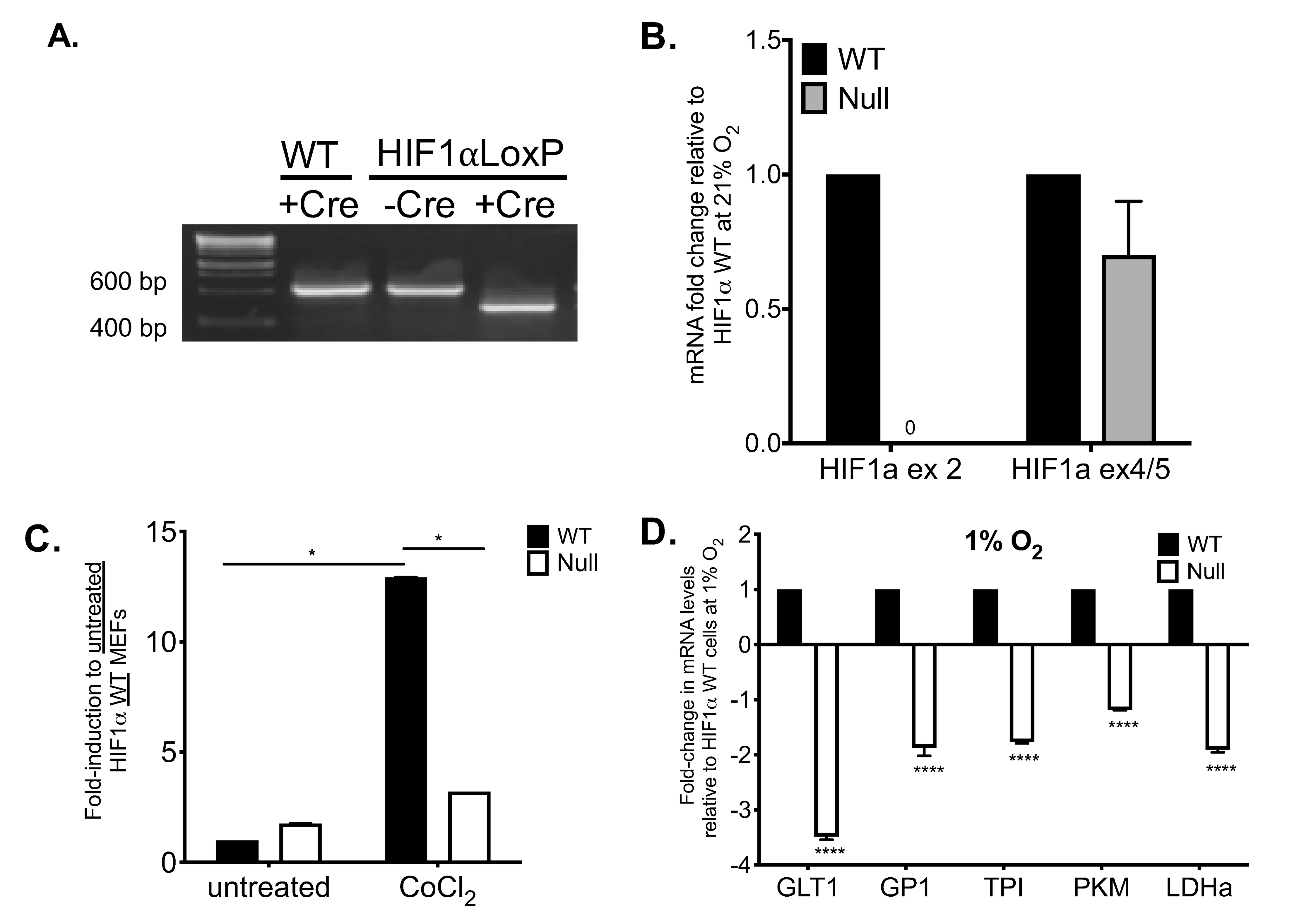

Supplement: S1 Fig — (A-D) Mouse embryonic fibroblasts (MEFs) were isolated from 13.5-day old embryo from B6.129-Hif1atm3Rsjo/J (HIF1αLoxP) and C57BL/6J (WT) and were immortalized by culturing cells over 30–35 generations. Immortalized HIF1αLoxP MEFs cells were transduced with a lentivirus vector expressing Cre-recombinase (Lenti-Cre) and selected with Blasticidin. MEFs (WT) isolated from parental mice was used as corresponding control for all experiments. (A) Excision of exon 2 was detected by amplification of gene fragment spanning exon 1 to exon 5 by PCR. A 400 bp fragment corresponds to the excised exon 2 in Null MEFs (+CRE) in comparison to 600 bp fragment (-CRE) in HIF1αLoxP MEFs. (B) HIF1α mRNA expression in WT and Null cells were measured by qPCR with primers from Exon 2 region. Exon 4/5 from HIF1α primer was used as corresponding control and was detected in both WT and Null cells. (C) WT and Null MEFs were either treatment with the hypoxic mimic cobalt chloride (CoCl2) to induce HRE-driven luciferase expression for 8 hours or left untreated. Data shown in graph is the average of three experiments performed independently with triplicates. Statistical analysis by Multiple Student’s t-test, mean ± SEM. *, p<0.05. (D) WT and Null MEFs were exposed to 1% O2 and HIF1 alpha target genes such as Glutamate transporter (GLT), Glucose-6-Phosphate Isomerase (GPI), Triose-phosphate Isomerase (TPI), Lactate Dehydrogenase A (LDHa), Pyruvate Kinase M1/2 (PKM) were measured by qPCR. ΔΔCt normalized against WT infection at 21% O2 and displayed as 2-ΔΔCt fold-change. Data shown in graph is the average of three experiments performed independently with triplicates. Statistical analysis by Multiple Student’s t-test, mean ± SEM. ****, p<0.0001. (TIF) [file ppat.1008192.s001.tif]

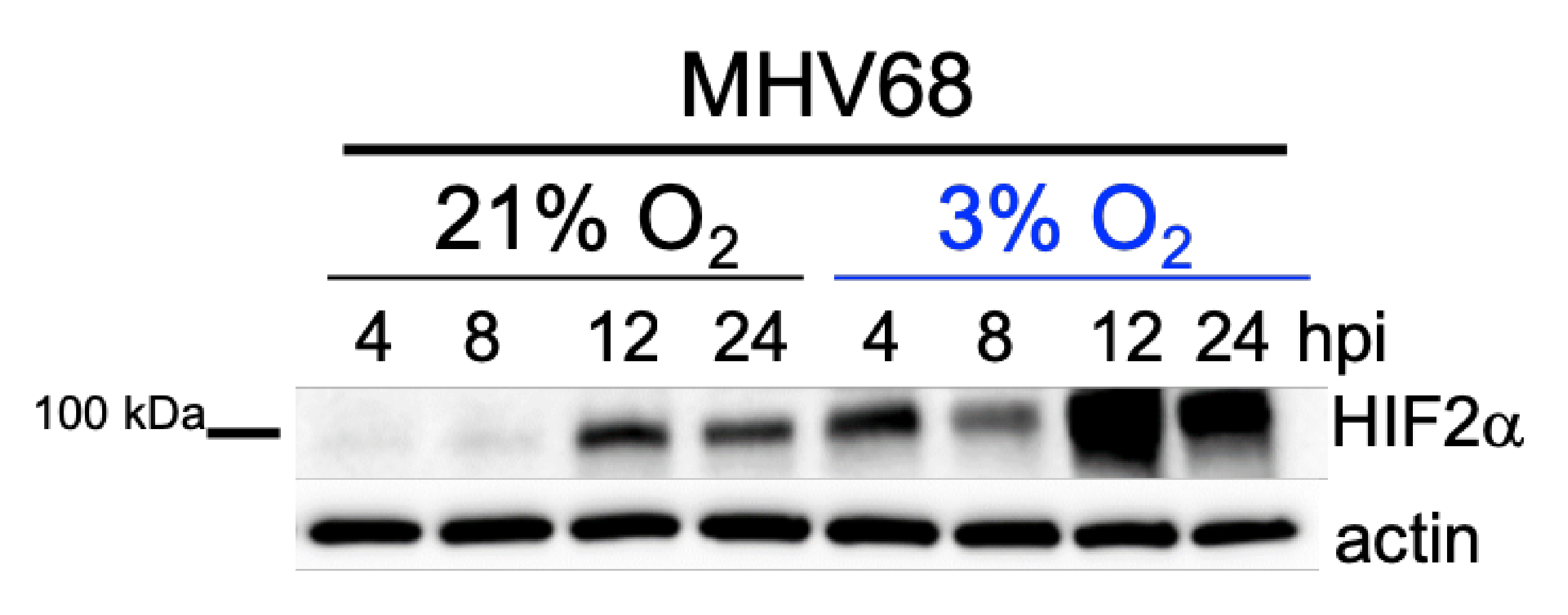

Supplement: S2 Fig — (A) 3T12 fibroblasts were infected with a wild type strain of MHV68 (WUMS) (5 MOI) at 21% O2 (cell culture incubator) and transferred to either 21% O2 and 3% O2. Protein lysates were analyzed by western blot for the expression of HIF2α protein at different time-points. Immunoblots are representative of three experiments performed independently. (TIF) [file ppat.1008192.s002.tif]

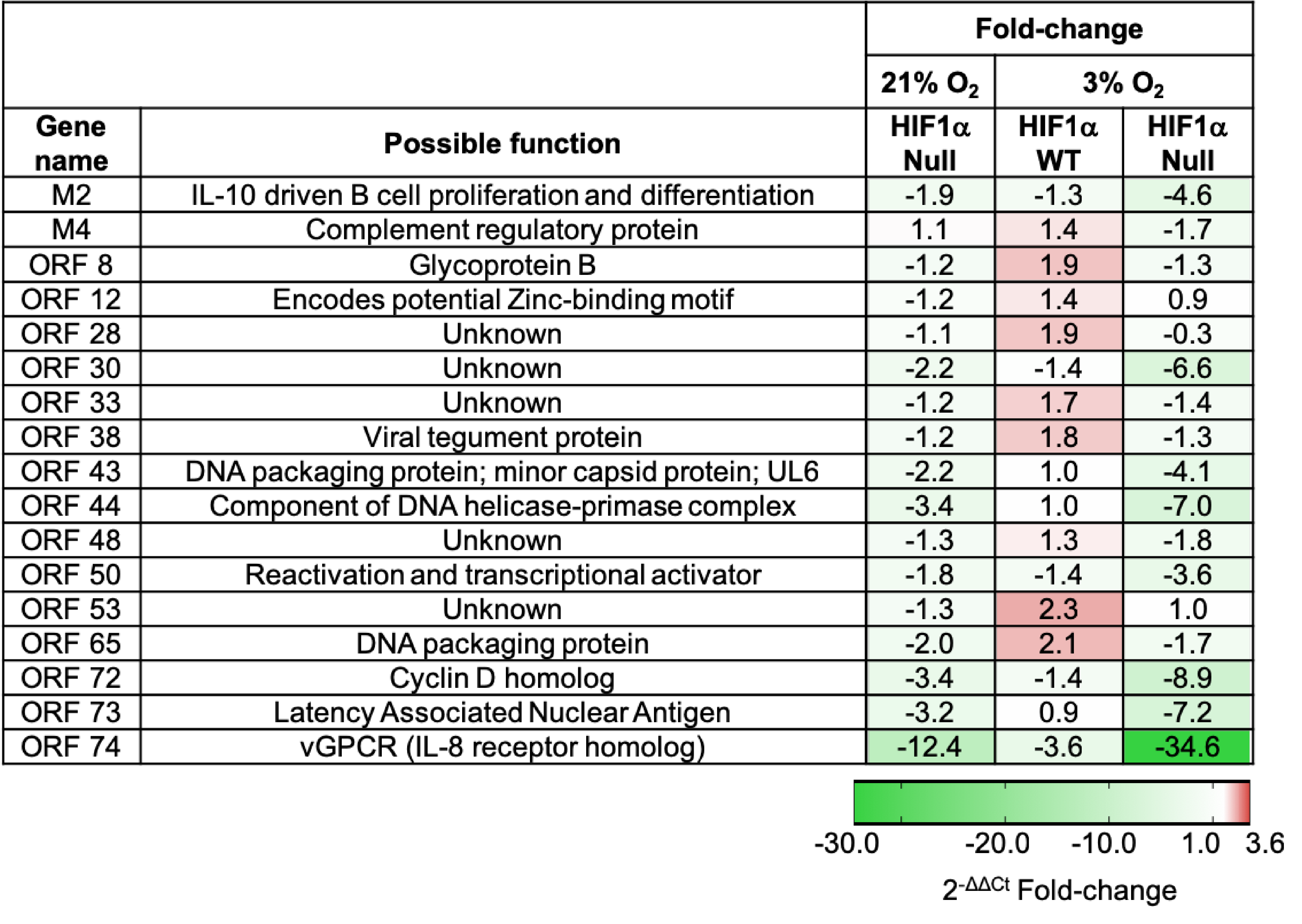

Supplement: S1 Table — HIF1α WT and HIF1α Null MEFs were infected with MHV68 (MOI 5.0) and transferred to either 21% and 3% oxygen, RNA was isolated 24 hpi. Levels of mRNA for MHV68 ORFs with HRE were determined by qPCR; ΔΔCt normalized against WT infection at 21% O2 and displayed as 2-ΔΔCt fold-change. Heat map was created using GraphPad Prism. Statistical significance displayed as asterisk (*, p<0.05) were determined using GraphPad Prism by Bonferroni’s multiple-comparison test as following: column 1: 21% O2 HIF1α Null vs 21% O2 HIF1α WT, column 2: 21% O2 HIF1α WT vs 3% O2 HIF1α WT, column 3: HIF1α Null vs 21% O2 HIF1α WT. (TIF) [file ppat.1008192.s003.tif]
