# Supplementary material for: A role of hypoxia-inducible factor 1 alpha in Murine Gammaherpesvirus 68 (MHV68) lytic replication and reactivation from latency
Source: PLoS Pathog. 2019 Dec 6;15(12):e1008192. doi: 10.1371/journal.ppat.1008192 (PMC6975554; doi:10.1371/journal.ppat.1008192)
Supplement: Attachment — Submitted filename: Response to Reviewers.pdf [file ppat.1008192.s004.pdf]

Reviewer 1

It was very reassuring that this reviewer thought "*the whole study revealed in vivo function of HIF1a in MHV68 replication and reactivation*". We thank this reviewer for his/her encouraging comments, analysis and feedback which has been essential to improve our paper.

1. *Fig1A, the band of HIF1a is weak, especially when there is a stronger non-specific band above. It would be more convincing if author could include positive control on the same gel.*

We now include western blot data for positive HIF1α protein expression by $CoCl_2$ treatment and is represented as a single band with anti-HIF1α antibody by Abcam.

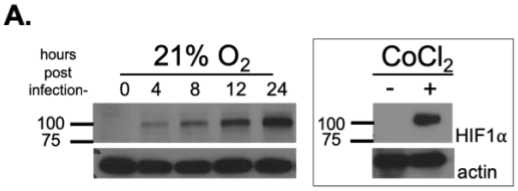

2. *Fig1D. Although the list of metabolic related genes are known to be induced by HIF1a, authors still need to confirm that the induction of these genes is dependent on HIF1a with MHV68 infection.*

Figure 3D comparing the expression of metabolic genes in presence or absence of HIF1α now shows that expression of metabolic genes by MHV68 is dependent on HIF1α activity.

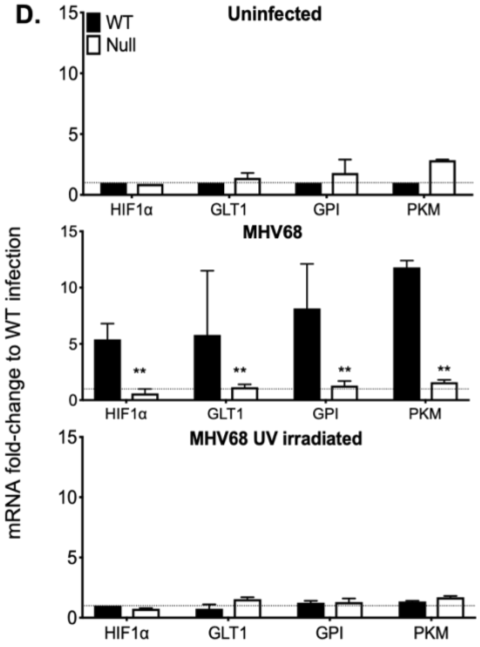

3. *In Fig3A, in HIF1a Null cells, hypoxia condition still strongly decreased viral production, implying factors other than HIF1a contributes to the reduction. Authors may provide some explanations, Similarly, ORF9 and ORF25 without HRE were strongly reduced in HIF1a Null cells under hypoxia, which is apparently non related to HIF1a.*

The goal of the experiment at 3% oxygen levels (now in Figure 4) was to show that in lower—yet physiological—oxygen level conditions in which upregulation of HIF1α was increased there was a stronger effect noticed when HIF1α was deleted. This is why these set of experiments are now showcased in Figure 4 for clarity. Regarding the regulation of non-HRE containing genes: in the current version of the manuscript we decided to include only HRE containing genes since—as now discussed in the current manuscript—we could not dissociate indirect effects due to the decreased transcription of genes because the viral gene regulating its expression was an HRE containing gene such as the case of the transactivator RTA.

4. *Fig. 5, authors showed the induction of glycolytic enzymes was abolished in HIF1a-Null cells with MHV68 infection, which may partially answer question #2 from this reviewer, but the result will be more convincing if authors would show that re-introduction of HIF1a can rescue expression of those genes in HIF1a-Null cells under infection."*

We appreciate this reviewer suggestion and idea. However, the nature of the HIF1α knock/out which deletes only Exon 2 (the DNA-binding Domain) precludes reconstitution as this truncated HIF1α lacking exon 2 could act as a dominant negative with WT HIF1α by competing for HIF1β heterodimerization

5. *It is a little choppy to study innate immune responses in Fig 5. IFIT8 was mentioned in the manuscript but not in the figures. Moreover, innate immune response is usually defined at very early time points post infection, however the authors detected those genes at 24 hpi.*

We agree with this reviewer's comment. The role of HIF1α in activation of innate responses during virus infection requires in-depth analysis outside the scope of this manuscript. To avoid diverging from the main focus of our study we have eliminated this section.

Finally, re-introduction of HIF1a will be very helpful to exclude the difference of WT and HIF1a Null cells.

See response to comment 4 above.

6. *Fig6B presentation of the data is confusing, if authors want to compare MHV68 WT vs MHV68-BAC, the authors may present WT vs BAC rather than show Cre virus here.*

We agree with the problem in the figure. In the current version we are providing better labeling for the scheme of animal infection.

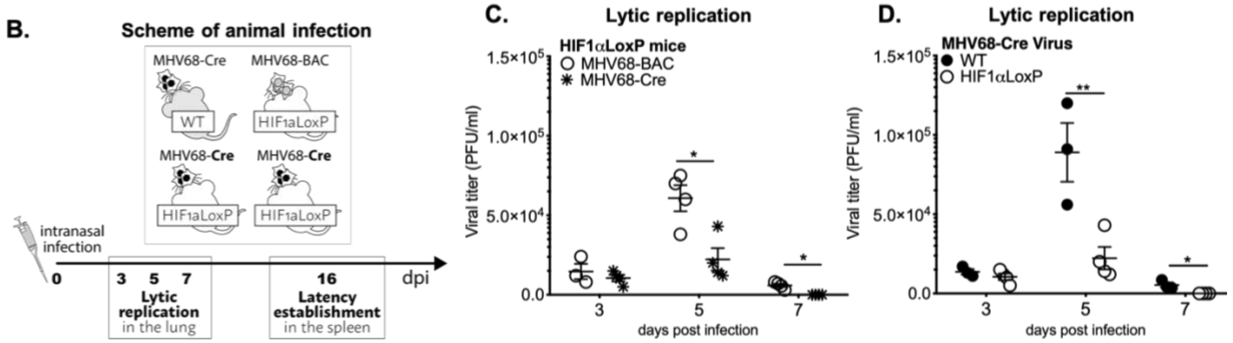

6D, it is also a little choppy here to show IL1b. Since authors present a series of IFNs in figure 5, why did the author show IL-1b production instead of IFNs in vivo? This reviewer also would like to remind the authors IL1b and TNFa at day 7 may not represent innate immune responses.

We have analyzed IL1β, IFNβ, IL-6, TNFα and IFNγ in lungs lysates on 3, 5 and 7 dpi but only IL1β levels in lungs from HIF1αLoxP mice showed decreased at day 7 when compared to WT infection. We also include an explanation for this result in the discussion. **Line 475: It is also likely that a reduction in viral expansion during the initial lytic phases in the lung could affect the extent of inflammation explaining the significant decrease of IL1β production in lungs lysates on day 7 (Figure 5D).**

7. *In Fig7B and D, reactivation in D16 and D42 was opposite. The authors explained in the discussion that the cell types are different. This reviewer fells that it is difficult to take the explanation, since the author already showed HIF1a contributes significantly to viral lytic replication. Did authors repeat Fig7D and get consistent results?*

In the original version there was not significant differences in latency establishment at day 42 and differences in reactivation were not very marked and very difficult to explain with do-able experiments. Therefore, we decided to focus and expand only on the results at Day 16 showing an impact of HIF1α deletion in reactivation which we complemented with the new Figure 7.

Reviewer 1 Minor issues (No response)

Reviewer 2

We appreciate that this reviewer found that our study *“adds new information in direct measurement of lytic virus infection and in vivo latency and reactivation using the mouse gammaherpesvirus system”.* We appreciate this reviewers’ criticisms and suggestions that have helped us to improve our manuscript.

1. *"Stabilization" of HIF1a protein is stated repeatedly, yet no experiments are presented that support the claim in this system. The conclusion needs to be excised from the paper or data should be added to support.*

We agree and appreciate this reviewer's remark and suggestion. Since the focus on the manuscript was not enquiring into the nature of the HIF1α upregulation induced by MHV68 infection we modified the conclusion of the experiments according to this reviewers' suggestion to accumulation/ increased levels etc.

2. *Demonstration of Cre efficiency and kinetics in the in vivo infections is important to understanding the effect of HIF1a deficiency on chronic infection. It is difficult to discern whether the effects on latency and reactivation are simply downstream consequence of replication defect or are specific to other states of infection. This is compounded by the fact that the in vivo experiments make use of Cre expressing virus infecting HIF1a floxed mice while the in vitro replications studies cell lines derived from fibroblasts already HIF1a deleted.*

We choose the HIF1α system vs Cre-virus *in vitro* because we found that Cre was expressed after HIF was upregulated by *de novo* infection. Although this was not the ideal solution, we found it was suitable for the needed experiments and that the results we obtained with the HIF1α Exon 2 deleted cells were compatible with the ones we obtained with the HIF1αLoxP animals and Cre-virus. To further support the validity of our in vivo Cre-induced deletion system, we now provide the proof of excision of HIF1α exon 2 by MHV68-Cre in splenocytes and lung tissues at day 16 **(now, Figure 6C)**

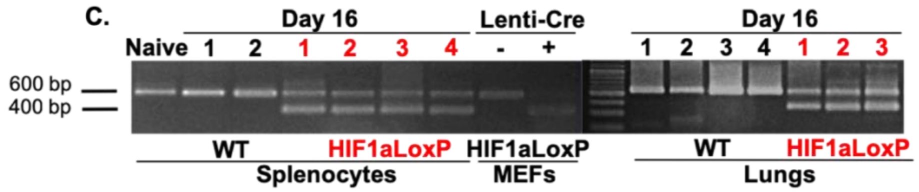

3. *Data analysis is not transparent: blanket statement assures that all experiments were conducted at least 3 times, but report does not state whether figures represent averages or representatives;*

We agree with the lack of consistency and accurate description pointed out by the reviewer. We now include in all figure legends how many times the experiment was conducted and whether the result represents an average or representatives.

comparison of infected cells to uninfected cells for luciferase activity is likely unfair because virus infection is likely to impact transcription at both specific and non-specific levels, better would be an internal control such as dual luciferase.

We agree with this reviewer on the importance of using Dual luciferase to gauge non - specific transcriptional effects of viral infection. We thank this reviewer that identified the lack of proper descriptions for our Dual luciferase experiments already done in the first manuscript. In this version we clearly state the use of dual luciferase assay was used in figures 1C and 2C.

Reviewer 2 Minor issues

1. *RTA data mentioned and listed in the figure legend was not included. Inclusion of additional viral gene expression profiles would also be a big improvement.*

   We agree that showing actual *in vivo* evidence of viral gene expression would strengthen the manuscript. Thus, we are now showing definitive evidence of MHV-68 Cre expression in the lungs by showing HIF1α exon2 deletion.

2. *Host cytokine response profile would benefit from greater context, such as discussion of other published studies or demonstration of expression and better justification for the small set analyzed in vivo.*

   Done in the discussion. We focalized in having at least a cytokine marker of inflammation (see answer to question 6 of Reviewer 1)

3. *Editorial improvements in grammar (commas, spelling) could be made.*

   We have address these in the attached manuscript.

Reviewer 3

We are glad that this reviewer found that our *"results support a role of HIF1a during MHV-68 infection both in vitro and in vivo".* We are thankful for this reviewer's comments that have been instrumental in improving our manuscript. We are particularly grateful for this reviewer's idea on using *ex vivo* reactivation in lower oxygen conditions which now constitute the new Figure 7 which provides further experimental support of Figure 6 results on the role of HIF1α in reactivation from latency.

1. *One major interesting observation is that MHV68 infection increases HIF1a protein at 8 hrs (Fig. 1A). However, the authors did not get into details about this increase. This is what was described in the paper, line 115-117, "HIF1a stabilization was observed until 24 hours, which was also correlated with a 6-fold increase in HIF1a mRNA levels (Fig1B)". This statement raises several questions. What evidence supports stabilization? There was no protein half-life comparison in this study. Does MHV68 affect VHL? The transcripts were examined at 24 hours. How much does transcription upregulation contribute to this protein level? What is the mechanism of HIF1a transcript and protein upregulation by MHV68? For comparison, un-infected under hypoxia should be as a control. In short, the mechanisms by which MHV68 infection up-regulates HIF1a need to be studied here.*

We thank this reviewer for his/her in-depth analysis and suggestions. Please see answer to question # 1 of Reviewer 2.

2. *Under both normoxia and hypoxia, virion production was reduced when functional HIF1a protein is absent (Fig. 3A). This data would indicate that HIF1a plays a positive role in MHV-68 replication. However, in WT cells under hypoxia (3% O2), where HIF1a protein is stabilized and allowed to perform its function, viral gene expression seemed to increase (Fig. 4), and yet virion production was reduced compared to 21% O2 (Fig. 3A). How do authors reconcile these pieces of data?*

We agree with the reviewer that there was a slight difference at 5 MOI and no difference at 0.5 MOI. However; the goal of the experiment at 3% oxygen levels (now in Figure 4) was to show that in lower—yet physiological—oxygen level conditions in which upregulation of HIF1α was increased there was a stronger effect noticed when HIF1α was deleted. This is why these set of experiments are now showcased in Figure 4 for clarity. In following with the new Figure structure and its content/ interpretation the current gene expression data represents fold decrease comparing HIF1α null to WT and as explained in the text showed a higher impact on HRE containing genes when the experiment was done in 3%.

3. *One potential issue for moderate phenotypes observed in vivo is due to the use of Cre-expressing MHV68 to knockout HIF1a. This does not eliminate the pre-existing HIF1a protein that is postulated to be stabilized by MHV68 infection. Authors should compare viral replication using the Cre-expression virus and HIF1a LoxP MEFs cells as in Fig.3A.*

We thank this reviewer for his/her in-depth analysis and suggestions. Please see answer to question # 2 of Reviewer 2

-Was ex vivo reactivation in Fig. 7B/7D performed under hypoxia?

We sincerely thank this reviewer's interesting idea, we have now generated data showing that low oxygen levels do not affect frequency of reactivation of latently infected splenocytes, however it accelerates virus replication and increases virus production significantly.

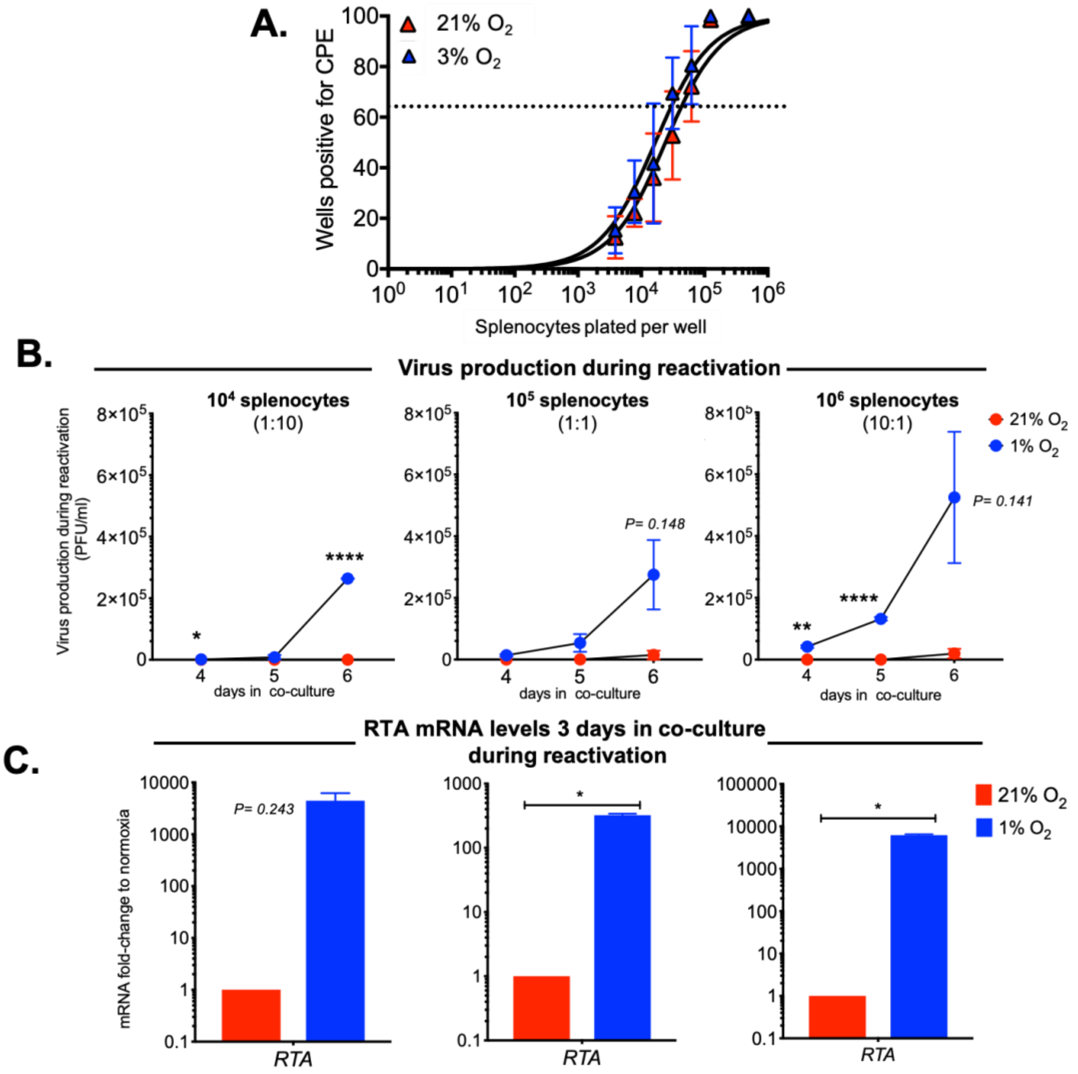

-And it is not clear whether latently infected cells already have HIF1a exon 2 removed or the removal happens during reactivation.

We have provided evidence for MHV68-Cre activity in latently infected splenocytes derived from day 16 infection by excision assay.

Reviewer 3 Minor issues:

1. *Fig5. There is no data IFIT8 (line 263). If there is little reduction of IFN-related genes in knockout cells, one would expect that viral replication increases in knockout cells both in vivo and in vitro. And yet the opposite was observed. Why?*

We have made edited the manuscript and IFN-related studies *in vitro* have been eliminated. Moreover, we provide explanation in the discussion as follows in **line 475, It is also likely that a reduction in viral expansion during the initial lytic phases in**

the lung could affect the extent of inflammation explaining the significant decrease of IL1β production in lungs lysates on day 7 (Figure 5D).

2. *Fig 6A&B, authors described how much reduction in viral titer (line 299-307) for day 3 and day 5 but did not give the numbers for day 7.*

We have included titers for day 7. **Line 318: The decline in viral titers continued until day 7 post infection with titer below limit of detection for HIF1αLoxP infection when compared to 5.4 X 10^3 PFU/ml ± 1.6 X 10^3 virus in WT mice (Figure 5C).**

3. *Fig. 7. Please label Null as MHV68-Cre.*

We apologize for the confusion. Now, a diagram for the scheme of animal infection clarifies labelling.
