# Supplementary material for: A role of hypoxia-inducible factor 1 alpha in Murine Gammaherpesvirus 68 (MHV68) lytic replication and reactivation from latency
Source: PLoS Pathog. 2019 Dec 6;15(12):e1008192. doi: 10.1371/journal.ppat.1008192 (PMC6975554; doi:10.1371/journal.ppat.1008192)
Supplement: Attachment — Submitted filename: ResponsesToReviewers.pdf [file ppat.1008192.s005.pdf]

Reviewer 1

We like to thank this reviewer for his/her comments, analysis and feedback. It is very encouraging this reviewer agrees that "*this study is significant in being the first one to document the in vivo importance of HIF1a in MHV68 replication and reactivation*".

1. *Fig. 1A and 1B and Fig. 4A – Do the authors know what happens to HIF-2alpha mRNA and protein in their experiments? Does infection with MHV68 affect the levels of HIF-2alpha as well as HIF-1alpha? If so, might HIF-2alpha be partially substituting for HIF-1alpha in experiments in which HIF-1alpha is mutated? Also, it would be nice to see the proteins from the experiments shown in Figs. 1A and 4A run on the same gel and probed concurrently so the reader than see the relative amounts of HIF-1alpha accumulated during virus infection under the two oxygen conditions.*

We appreciate this reviewer's comment. The modified manuscript includes HIF1α protein levels ran on the same gel to show relative amounts during infection at 21% O_2 vs 3% O_2.

Figure 4

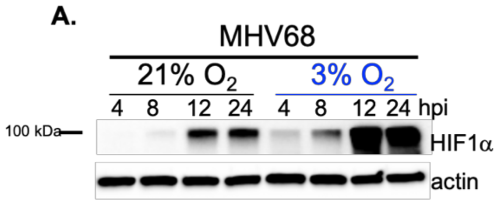

> Page 14, lines 307-309: In Figure 4A (top), we show that infection at low levels of O_2 upregulates HIF1α by 12hpi, sooner that normoxic infection (Fig. 1A) and uninfected cells cultured at 3% O_2 (Fig. 4A-bottom).

We found that indeed, and as described for KSHV, MHV68 infection upregulates HIF2α. However, we also found that this is hampered in cells missing HIF1α. Therefore, although it is plausible that the presence of low levels of HIF2α may mask the effects of HIF1α KO it is unlikely that in this scenario HIF2 would play a compensatory role as 1) cells do not appear compensate HIF1α loss with upregulation of HIF2α and 2) its upregulation during MHV68 infection appears to be, in part, HIF1α-dependent.

Supplementary Figure 2

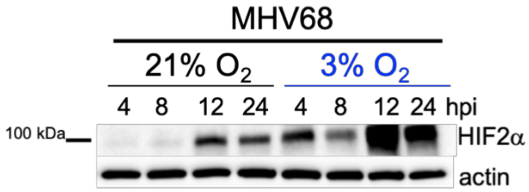

This insight is now included in the discussion as follows:

> Page 24, lines 520-531: As shown in Supp. Fig 2 and reported for KSHV infection [35], MHV68 does upregulate HIF2α. This observation precludes a possible role for HIF2α in compensating for HIF1α loss and masking the transcriptional consequences of HIF1α exon 2 deletion.

2. *Fig. 3A versus corresponding text on page 9 – Couldn't the minor, largely non-statistically significant differences observed here be due to the HIF-1alpha null MEFS simply being slightly less infectable with the virus, i.e., the real MOIs were not the same in the two cell strains? The authors responded to the reviewer #1's suggestion for a HIF-1alpha addback experiment by stating, "the nature of the HIF1alpha knockout which deletes only Exon 2 (the DNA-binding Domain) precludes reconstitution as this truncated HIF1alpha lacking exon 2 could act as a dominant negative with WT HIF1alpha by competing for HIF1beta heterodimerization". While this could happen, one may be able to circumvent this potential problem by adding back HIF-1beta along with HIF-1alpha to overcome the mutant HIF-1alpha protein potentially squelching HIF-1beta (although it might still squelch up co-activators of the HIFs). It would be worth giving this experiment a try.*

We appreciate this reviewer's comments. In this submission, to rule out any confounding effect due to long term effects of HIF1 deletion we carried out two alternative inhibition approaches A) we carried out a transient HIF1 siRNA silencing that showed the same replication phenotypes than the K/O B) We use a HIF1a specific inhibitor PX-478 that acts by decreasing HIF1a expression.

Figure 3

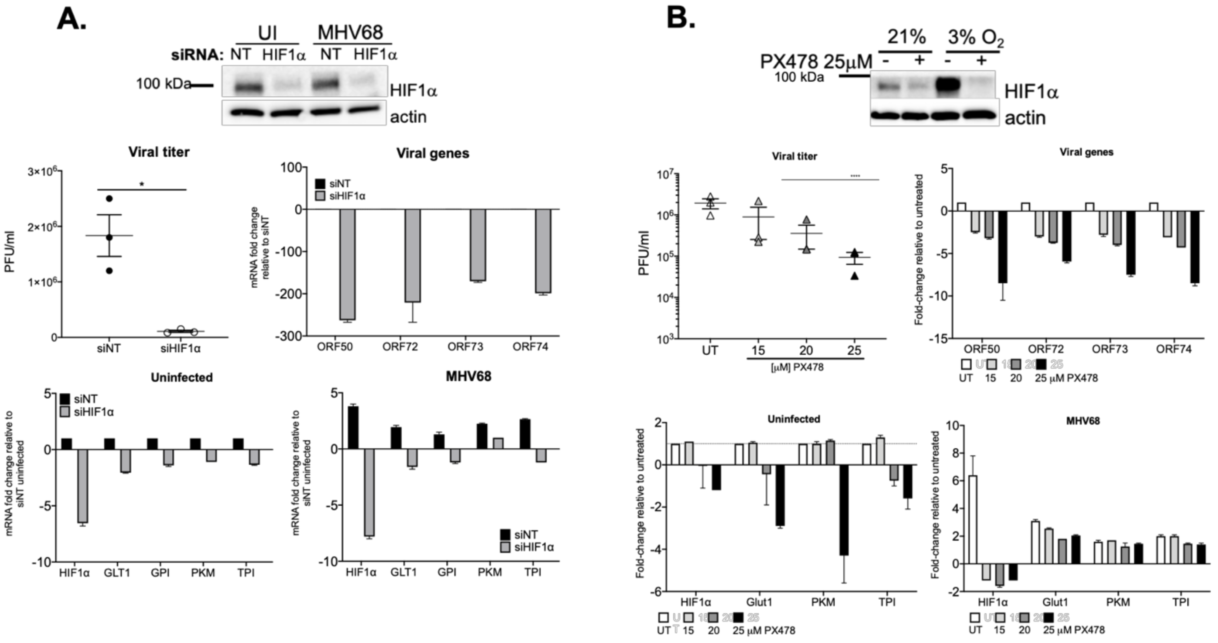

Pages 12-13, lines 270-290:

siRNA silencing and drug-mediated inhibition of HIF1α impairs MHV68 replication

In order to rule out any confounding effects due to the long-term impact of HIF1α exon 2 deletion in Null cells, we carried out two alternative approaches to inhibit HIF1 activity during MHV68 lytic infection. First, 3T12 cells were transfected with a HIF1α siRNA for 24 hours, followed by infection in normoxic conditions. The top panel of Figure 3A confirms HIF1α protein expression is abolished in HIF1α siRNA cells of uninfected and MHV68 infected cells cultured at 3% O_2. Silencing of HIF1α during normoxic infection significantly reduces viral titers by 20-fold at 48hpi, on average, and drastically downregulates the expression of lytic replication genes (Fig. 3A- 2nd row). In the second approach (Fig. 3B), we utilized PX478, a small molecule inhibitor that has been shown to potently inhibit HIF1α transcription activity [38], in addition to reducing HIF1α protein and mRNA synthesis [12]. In the first row of Figure 3B, we show 3T12 cells exposed to 25μM of PX478 decrease in HIF1α expression 24hpi even HIF1α-induced conditions. After MHV68 incubation, infected 3T12 cells were treated with 15, 20, and 25μM of PX478 and cultured at 21% O_2. At 48 hours, viral titers in supernatants from 25μM PX478 were 10-fold less than titers from untreated supernatants. Moreover, the extent of the downregulation of lytic genes, 24 hours prior, was parallel with the increment in PX478 concentration (Fig. 3B- 2nd row). Finally, blocking HIF1α activity through these approaches also impaired MHV68-induced expression of glycolytic genes (Fig.3- 3rd row). These data confirm our observations pointing to a critical role for HIF1α in MHV68 lytic replication.

3. *Fig. 3B - The authors note the existence of PUTATIVE HREs (based upon sequence) in the promoter regions of numerous MHV68 viral genes. However, the increased level of HIF-1alpha observed following infection with the virus also affects expression of some non-HRE-containing genes, presumably indirectly via cellular signaling pathways. It would be nice to document whether some of the key viral putative HREs identified here are truly functional HREs, e.g, by performing transient transfection assays with some of these HRE-containing promoters linked to a luciferase reporter.*

We greatly appreciate this reviewer's comments. We have included luciferase reporter activity of MHV68 ORF74 promoter region co-transfected with full-length HIF1α insensitive to oxygen-degradation.

Figure 2

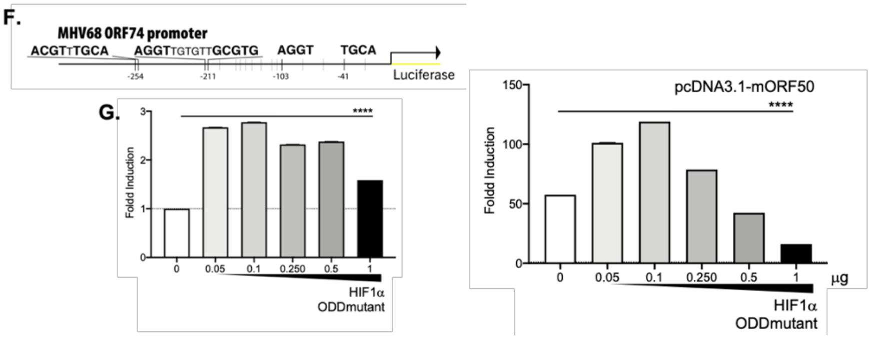

Page 12, lines 255-268:

The vGPCR (mORF74) viral promoter of MHV68 contains hypoxia responsive elements and is transcriptionally activated by HIF1α expression

Downregulation of ORF74 mRNA in HIF1α Null cells (Fig. 2D) and the presence of HREs consesus (Fig. 2F, ACGTG, AGGTG, GCGT) within this promoter point to a role for HIF1α in transcriptional regulation of the viral gene. To determine HIF1α dependent transcription activation, the promoter region spanning nucleotides at -597 to start codon of ORF74 was inserted upstream of the luciferase reporter pGL2-Basic vector. MHV68 ORF74 promoter luciferase construct was transiently transfected into 293AD cells with increasing amounts of an oxygen-degradation insenstitive HIF1α mutant. Figure 2G shows statistically significant 2.6-fold activation to mock transfection. Moreover, the addition of expression vector containing full-length MHV68 RTA (mORF50) further enhances promoter activity (2-fold) in the presence of constitutively active HIF1α. These findings suggest a role for transcription regulation of MHV68 ORF74 by HIF1α as previously observed in KSHV vGPCR [37].

4. *The new data presented in Fig. 6C indicate that only about 2/3rds of the HIF-1alpha genes contain the exon 2 deletion by day 16 after infection. Is that due to the preparations including some uninfected cells? What percentage of the HIF-1alpha genes are still wild-type at earlier times after infection? One really needs to look at the kinetics as well as efficiency of creation of the deletion in vivo given the virus-infected cells could be establishing latency when HIF-1alpha is present at a high level prior to loss of the wild-type HIF-1alpha gene, mRNA, and protein. In this case, similar efficiencies of establishment of latency could be a trivial consequence of HIF-1alpha protein still being present in the cells during those key early events.*

We appreciate this reviewer's question. Figure 6C represents amplification of HIF1α gene lacking exon 2 of bulk splenocytes which includes uninfected cells. We were not able to detect exon 2 deletion during earlier times of infection of lungs at day 3, 5 and 7 but following the splenic latency establishment period.

Reviewer 1 (Minor Issues)

1. *Fig. 1C and 2C – Y-axis is mean % relative to what? Also, the reporters used in these experiments are clearly described in the Methods section, but too cryptically mentioned in these figure legends to be understood. Please reword.*

We thank this reviewer for their comment on this issue. Results showing HIF1α-dependent transcription activity by MHV68 infection in 3T12 (Fig.1C) and its reduction in MEFs HIF1α Null is expressed as fold-induction to uninfected (Now Supplementary Figure 1C) and to MEFs HIF1α WT at 21% O_2. We have corrected the y-axis in each luciferase reporter figure.

2. *Fig. 2D – What is the y-axis? Log base 2?*

We thank this reviewer for the throughout examination our manuscript. The *y*-axis in this figure (now as Supplement Figure 1) describes fold-change of mRNA levels relative to MEFs with intact HIF1α gene, calculated by negative Log base 2 of delta delta Ct values. This has been corrected in the corresponding figures legends in the modified manuscript.

3. *Fig. 3A, Fig. 4B, Fig. 4C, Fig. 5C, Fig. 5D, and Fig. 7B – The tick marks on the y-axes correspond to log base 10, yet the labels on the large ticks differ by linear amounts. Is it the latter given most of the differences are rather small and some are not statistically significant? Please redraw the tick marks for these y-axes to be appropriate ones.*

We appreciate the attention to detail from this reviewer, agree and therefore eliminated the log tick marks in every figure mentioned above. The linear scale was selected to display the differences in viral production in comparison to wild-type HIF1α cells.

4. *Lines 973-975 (legend to Fig. 6B) – Are the numbers in parentheses reversed here?*

We thank this reviewer for offering a very detail evaluation. The numbers were reversed and have now been corrected in the figure legend.

5. *Were all of the animal experiments performed on 3 separate occasions with the data shown being one typical result? If not, please clearly indicate in the figure legends how many times EACH experiment was performed.*

We apologize for the misunderstanding. All experiments were performed at least three times with their respective technical replicates and it has been corrected in the figure legends of the manuscript.

6. *Please provide catalog numbers in Methods section for each antibody used here.*

Catalog numbers for all antibodies are now included in the Methods section.

> Page 30, lines 668-670: Recombinant Rabbit Anti HIF1α antibody (ab179483, Abcam), Anti-actin antibody (A5316, Sigma) and Recombinant Rabbit Anti-HIF2α (Novus Biologics, NB100-122).

7. *It would be nice to understand the mechanism by which infection with MHV68 increases HIF-1alpha levels, but that could be the subject of a follow up study.*

We thank this reviewer for their question. We agree in that the mechanism in which HIF1α is accumulated by MHV68 infection should be addressed in future studies.

Reviewer 2

1. *The authors were responsive to providing experiment numbers and representative or averaged data designations for each experiment. However, several experiments (Fig 1B/C/D, Fig 2D, Fig 3C, Fig 4B, Figu 5E are listed as "One experiment performed in triplicate", which if taken at face value, means that the experiment was carried out only once, with technical triplicates. If so, it is not appropriate to carry out statistics on the data, as SEM of technical replicates is artificially tight and therefore overestimates significance. Clarification is needed on this.*

We would like to thank the reviewer for their inquiry. Results in graphs from figures 1, 2, 3 and 4 represent the average of three experiments performed independently with triplication. For figure 5, graph represent one experiment (n= 3-4 mice) of three independent experiments, with similar viral titer differences. Statistical analysis was performed in Graph Pad Prism by Multiple Student's-t-test.

Reviewer 2 (Minor Issues)

1. *In response to Reviewer 2, point #2, the authors note that in lytic infection, HIF was induced prior to Cre expression but it does not appear that this is included in the manuscript. I suggest that this be included in the manuscript, whether in text or data in supplement. The reason for this is that the timing of Cre deletion in lytic infection is a matter of interest in a number of ongoing studies and publication of this and other observations are important to those using and considering this strategy.*

We agree with this reviewer in that this technical issue should be mentioned as it may also benefit research employing similar tools. We added a paragraph in the discussion regarding the choice for *in vitro* systems.

> Pages 21-22, lines 475-480: As a first approach we employed the MHV68-Cre infection of primary MEFs from the HIF1αLoxP mouse strain to address the consequences of HIF1α deletion in lytic infection. However, we noticed that Cre-induced deletion was detectable only after 12 hpi (data not shown) while MHV68 infection upregulates HIF1α well before--between 4 and 8 hours (Fig. 1A). Therefore, we resorted to create stable null cells and complement it with two other alternative inhibitory approaches.

Reviewer 3

1. *Figure 1C: KSHV infection and likely MHV68 upregulates almost any promoter in transient transfection assays, therefore, there needs to be a HRE mutant control for this experiment to be meaningful.*

We thank this reviewer for their recommendation and accordingly we have repeated the HRE-luciferase assay in Figure 1C to include background activity in HRE consensus mutated promoters from ACGT to TTGT.

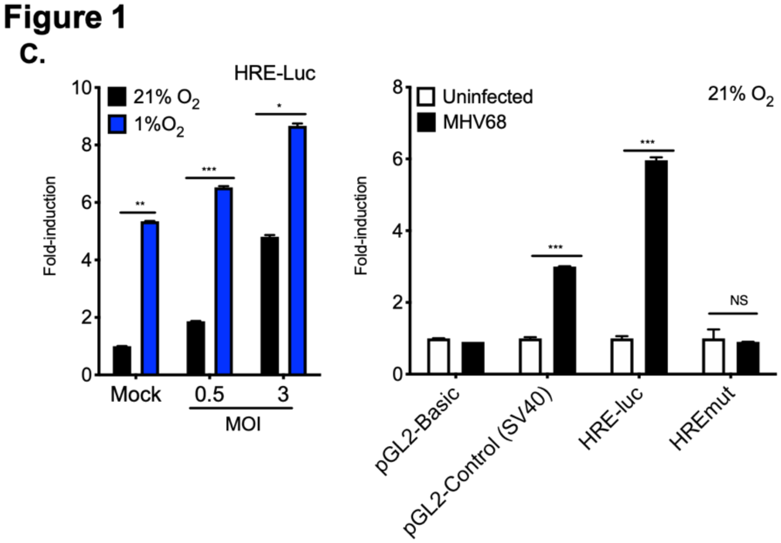

Page 7, lines 149-151: Substitution of HRE consensus nucleotides ablated luciferase response under MHV68 infection indicating HRE-dependent specific activation (Fig. 1C-right).

2. *There is an issue with the Y-axis scale on figure 3A (and again in 4B and C and figure 5C, D and E and 7B). They show a linear scale but the Y-axis has log10 marks on it making it seem more like a log scale. As titer is usually shown on a log scale this is misleading and should cleaned up. Unlike 7B, 7C has these marks and is actually a log scale so they should be left in 7C.*

We thank this reviewer for suggesting these changes. The figures mentioned above are corrected in the affixed manuscript and now contain the expected tick marks.

3. *For figure 3A, they state that at low MOI there are significant differences at later time points and they can only accurately state significance at a single time point, 72 hours.*

We admit to this reviewers' comment and made changes as follow:

> Page 9, lines 198-202: As shown in Figure 2B, time-course infection of Null cells at 5.0 MOI showed a slight reduction while a lower infection of 0.5 MOI had a significant decreased in virus production at later time-points. These results suggest a role for HIF1α during lytic replication. Thus HIF1α is necessary for efficient production of infectious particles during MHV68 replication.

4. *On line 227 they state that "Taken together our results indicate that virally-induced HIF1 MAY directly or indirectly participate in the expression of many HRE-containing and non-containing promoters regulating early and late genes…." However, the data only show that these genes are down regulated. It could be that the absence of HIF prevents ORF50 alone which would block almost all of the other promoters and therefore, HIF1 does not play a direct role and this is a gross overstatement. This is mentioned in the discussion but is misstated here.*

We are thankful to this reviewer for their recommendation. This sentence now read as follows:

> Page, 11 lines 231-234: Taken together our results indicate that HIF1α may directly or indirectly regulate expression of HREs-containing viral genes required for optimal growth kinetics during MHV68 replication.

5. *Figure 5E represents the only pro-inflammatory cytokine that showed significant effects while all others tested did not. Is this really indicative of inflammation in the lung?*

We agree with this reviewer. Results stated for this figure now read as follow:

> Page 17, lines 374-376: The reduction in IL1β on day 7 post-infection in the absence of HIF1α activity may be due to early viral clearance reflected titers at day 5.

6. *In figure 7B they show that there is a significant difference in the amount of virus produced from reactivation in low oxygen conditions. In figure 5D they perform a similar experiment with de novo infection of cells but they set the wild type cells in both normoxia and hypoxia to 1. It might be useful to show if the level of gene expression in wild type cells is altered by hypoxia as the data must already be there and would corroborate the data in 7B and C nicely. If not, they should explain why there is not a change.*

We are grateful to this reviewer's guidance. We now include average fold-change of viral mRNA in wild-type infection at 3% O_2, normalized to 21% O_2 (normoxia) as shown in supplementary table 1. The data and statement are displayed as follow:

Supplementary Table 1

		Fold-change		
		21% O_2	3% O_2	
**Gene name**	**Possible function**	**HIF1α Null**	**HIF1α WT**	**HIF1α Null**
M2	IL-10 driven B cell proliferation and differentiation	-1.9	-1.3	-4.6
M4	Complement regulatory protein	1.1	1.4	-1.7
ORF 8	Glycoprotein B	-1.2	1.9	-1.3
ORF 12	Encodes potential Zinc-binding motif	-1.2	1.4	0.9
ORF 28	Unknown	-1.1	1.9	-0.3
ORF 30	Unknown	-2.2	-1.4	-6.6
ORF 33	Unknown	-1.2	1.7	-1.4
ORF 38	Viral tegument protein	-1.2	1.8	-1.3
ORF 43	DNA packaging protein; minor capsid protein; UL6	-2.2	1.0	-4.1
ORF 44	Component of DNA helicase-primase complex	-3.4	1.0	-7.0
ORF 48	Unknown	-1.3	1.3	-1.8
ORF 50	Reactivation and transcriptional activator	-1.8	-1.4	-3.6
ORF 53	Unknown	-1.3	2.3	1.0
ORF 65	DNA packaging protein	-2.0	2.1	-1.7
ORF 72	Cyclin D homolog	-3.4	-1.4	-8.9
ORF 73	Latency Associated Nuclear Antigen	-3.2	0.9	-7.2
ORF 74	vGPCR (IL-8 receptor homolog)	-12.4	-3.6	-34.6

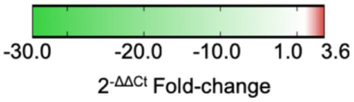

Page 15, lines 325-327: Levels of mRNA expression of some MHV68 HRE-containing genes were modestly increased during wild-type infection at 3% O_2 of HIF1α WT cells (Supplementary Table 1).

Reviewer 3 (Minor Issues)

1. *The section title on line 232 is not accurate. These genes have not been shown to affect MHV68 replication, only the replication of other viruses so this statement should not be made.*

We would like to thank this reviewer and have agree on the following title for this section:

Page 11, line 235:
HIF1α activity is required to induce host genes during MHV68 replication

2. *Line 434: the sentence does not make sense as written. "In searching for possible explanations for the decrease in viral replication, we analyzed the expression of viral HRE containing genes (Fig. 3B) and found that in 7 of 17 viral gene mRNA levels were decreased in the HIF deleted (Fig. 3C)."*

Based on this reviewers' comment we revised the sentence and it reads as follows:

Page 21-22, lines 479-482: We found that deletion of the DNA-binding motif in Null cells impaired viral replication (Fig. 3A) and gene expression analysis of

MHV68 viral HRE-containing promoters reveal an effect in 7 of 17 ORFs (Fig. 2D, 2G and Fig. 3).

3. *Line 450: This is an extremely long dense sentence*

We agree with this reviewer's suggestion and have made the following changes to the structure of this sentence:

> Page 22, lines 496- 499: Previous results published by our laboratory showed that the glycolysis inhibitor 2-deoxyglucose (2DG) inhibited MHV68 lytic infection which is consistent with our results pointing to a role of HIF1α regulation of glycolytic genes as part of gammaherpesviruses strategy to reprogram glucose metabolism needed for replication.

We have addressed the submission requirements following a message from the Plos Pathogens staff. We have removed financial details from the Acknowledgements section. We have corrected the statement regarding funders role in the development of the manuscript. In addition, we have removed the author contributions from the acknowledgements section and have added them to the Author Contributions section under “Edit Author Details” in the “Add/Edit/Remove Author” section of the submission form. Finally, we have eliminated the use of “data not shown” in the manuscript.
